# A prospective analysis of risk factors for pediatric burn mortality at a tertiary burn center in North India

**DOI:** 10.1186/s41038-017-0095-7

**Published:** 2017-09-20

**Authors:** Amol Dhopte, Rahul Bamal, Vinay Kumar Tiwari

**Affiliations:** 1 Department of Plastic, Reconstructive and Maxillofacial surgery, Government Medical College and Hospital, Nagpur, 440003 India; 2 Department of Burns, Plastic and Maxillofacial Surgery, VMMC & Safdarjung Hospital, New Delhi, India; 3 Department of Burns and Plastic Surgery, PGIMER & RML Hospital, New Delhi, India

**Keywords:** Pediatric burns, Mortality, Risk factors, Microbiological cultures, India

## Abstract

**Background:**

None of the available mortality predicting models in pediatric burns precisely predicts outcomes in every population. Mortality rates as well as their risk factors vary with regions and among different centers within the regions. The aim of this study was to identify socio-demographic and clinical risk factors for mortality in pediatric burns in an effort to decrease the mortality in these patients.

**Methods:**

A prospective analytical study was conducted in patients up to the age of 18 years admitted for burn injuries in a tertiary care burn center in India from January to December 2014. Clinical and demographic data was collected through questionnaire-interview and patient follow-up during their stay in the hospital. Univariate and multivariate firth logistic regression was used to identify various risk factors for mortality in pediatric burns.

**Results:**

A total of 475 patients were admitted during the study period. Overall mortality was 31.3% (*n* = 149) in this study. Mean age of the patients who died was 8.68 years. Of the 149 deaths, 74 were males and 75 were females (male to female ratio = 0.98). Mean total body surface area (TBSA) involved of the patients who expired was 62%. Inhalational injury was seen in 15.5% (*n* = 74) of pediatric burn admissions. Mortality was significantly higher (74.3%) in patients with inhalation injury. Mortality was highest in patients with isolates of Acinetobacter + Klebsiella (58.3%), followed by Pseudomonas + Klebsiella (53.3%), Acinetobacter (31.5%), and Pseudomonas (26.3%) (*p* < 0.0005). Factors found to be significant on univariate firth analysis were older age, female gender, suicidal burns, higher TBSA, presence of inhalation injury, increased depth of burn, and positive microbial cultures. On multivariate analysis, higher TBSA was identified as an independent risk factor for mortality. The adjusted odds ratios for TBSA involvement was 21.706 (25.1-50%), 136.195 (50.1-75%), and 1019.436 (75.1-100%), respectively.

**Conclusion:**

TBSA is the most important factor predicting mortality in pediatric burns. The higher the TBSA, the higher is the risk of mortality. Other significant risk factors for mortality are female gender, deeper burns, positive wound cultures, and inhalation injury. Risk of mortality was significantly lower in children who belonged to urban areas, nuclear family, who sustained burn injury in the last quarter of the year, and who stayed in the hospital for longer period.

## Background

Burn injuries are common occurrences in India, and pediatric burn injuries constitute a substantial part of this group of patients [1]. India has a large pediatric population, and pediatric burns represent 17–25% of total burn admissions [1, 2]. Mortality rates for pediatric burns as well as their risk factors vary with regions and among different centers within the country. Various studies from India reported a mortality rate of 7–12% [1–3].

Over the years, survival in pediatric burns has improved world over, but the situation in India is different. The most important variables directing patient outcomes in our country are delay in arrival at a burn facility from remote villages, lack of early wound coverage, and sepsis. Burn wound infection and sepsis are still the most significant factors causing mortality in pediatric burn patients as shown by various studies [4, 5].

Models for predicting mortality in pediatric burn patients are available in abundance. Age, burn size, and inhalational injury are variables used in every one of them. Commonly utilized prediction models for pediatric burns mortality are Baux scores, Abbreviated Burn Severity Index (ABSI) score and Clark’s model [6–9]. None of these models precisely predict outcomes universally in all populations [10].

Worldwide, there are a number of studies that predict the risk factors for burn injuries and burn related mortality in children [11–13]. However, to the best of our knowledge, there is no published study from India identifying the risk factors for mortality in pediatric burn patients. The aim of this study was to identify various socio-demographic and clinical risk factors for mortality in pediatric burn patients that can guide us in our endeavor to decrease mortality in this subset of our population.

This study was conducted at the department of Burns, Plastic and Maxillofacial Surgery, Safdarjung Hospital, New Delhi. Safdarjung Hospital has the largest tertiary care burn center in the country. It receives patients mainly from Delhi and neighboring North Indian states. Being a tertiary care burn center, both old and new burn cases are referred to this hospital. Referring hospitals include primary and secondary health centers along with tertiary level private hospitals. This dedicated burn unit is being managed by plastic surgeons and consists of 15-bed Burn Intensive Care Unit (BICU), 17-bed stepdown intensive care unit, 32-bed general burn ward, burns operation theater, and a physiotherapy unit.

## Methods

This was a prospective analytical study that included all the burn patients up to the age of 18 years admitted in the hospital from January to December 2014 [14]. Admission criteria included total body surface area (TBSA) involvement of more than or equal to 10%, involvement of face, hand, perineum and signs of inhalation injury. Questionnaire-interview was undertaken for all patients to obtain data regarding demographics and circumstances of injury. Thorough clinical assessment of the patient was done at the time of admission. These exercises of data collection were conducted by the doctor on duty.

The etiological classification of burn injuries used at our institute is thermal burn, scalds, electric burn, and chemical burn. Thermal burn denotes burn injuries sustained due to dry heat (e.g., flame burns, fire cracker burns, and contact burns), while scalds are burn injuries due to moist heat (e.g., burn injury due to steam, hot oil, hot water etc.) [15, 16] Lund and Browder charts were used for rapid assessment of involved TBSA. Signs of inhalation injury include increased respiratory rate, hoarseness, being burned in an enclosed space, altered mental status, head and neck burns, singed nasal hairs, inflamed oral mucosa, and carbonaceous sputum [17].

Patients were treated in the burn unit as per the standard protocol followed at the burn center. After initial resuscitation and stabilization, burn wound is dressed under sterile condition in the casualty dressing room by the plastic surgery resident. All pediatric burn patients with partial thickness burns are covered with collagen dressings. Topical 1% silver sulphadiazine cream is used in patients with infected collagen dressings and in patients with deep burns. Silver dressings are used in deep, exudating or infected wounds, subject to availability in the hospital. All burn wounds are given bulky dressings using Gamgee pads and roller bandages. Split thickness skin grafting is done for coverage when healthy granulation tissue appears over the burn wound.

Wound swabs for culture and sensitivity were from an area showing signs of infection on day 5 post-burn. Local signs of infection include conversion of a partial-thickness to full-thickness injury, worsening cellulitis of surrounding normal tissues, eschar separation, and tissue necrosis (American Burn Association) [18].

### Statistical analysis

Categorical variables were presented in number and percentage (%), and continuous variables were presented as mean ± SD or median. Normality of data was tested by Kolmogorov-Smirnov test. If the normality was rejected, then non-parametric test was used. Quantitative variables, i.e., age, body area involved and family size were compared using Mann-Whitney test (as the data sets were not normally distributed). Qualitative variables were correlated using chi-square test/Fisher’s exact test. Univariate and multivariate firth logistic regression was used to assess the association of mortality with various parameters. A *p* value of <0.05 was considered statistically significant. The data was entered in MS Excel spreadsheet, and analysis was done using Statistical Package for Social Sciences (SPSS) version 21.0. Firth logistic regression analysis was done using R software.

## Results

Of the 475 pediatric burn patients admitted during the study period, 59.1% (*n* = 281) were males and 40.8% (*n* = 194) were females. Majority of the patients belonged to 1–5 years of age group (50.1%). There were 80.21% (*n* = 381) patients belonging to nuclear family and 69.89% (*n* = 332) residing in rural areas. The mean TBSA involved was 37% ± 24%, and the median TBSA was 30% (inter quartile range: 20 to 50%). Totally, 76.6% (*n* = 364) patients had TBSA involvement of 10–50% while 23.3% (*n* = 111) patients had TBSA involvement of more than 50%.

A total of 326 wound swabs were sent in 326 patients for culture of microorganisms from the burnt wound. Various microorganisms isolated from these samples were *Acinetobacter baumannii* (*n* = 73; 22.4%), *Pseudomonas aeruginosa* (*n* = 38; 11.7%), *Staphylococcus aureus* (*n* = 26; 8.0%), *Klebsiella species* (*n* = 28; 8.6%), *Acinetobacter *+ *Klebsiella* (*n* = 12; 3.7%), *Pseudomonas* + *Klebsiella* (*n* = 30; 9.2%), *Escherichia coli* (*n* = 18; 5.5%), methicillin resistant *Staphylococcus aureus* (MRSA) (*n* = 3; 0.9%), and *Clostridia* species (*n* = 2; 0.6%). No growth was reported from 62 samples (19%).

### Factors affecting mortality

Distribution of various parameters between deceased and survivors are detailed in Table 1. Of the 475 patients admitted during the study period, 149 patients (31.3%) expired and 326 patients (68.6%) survived. The mean age of patients who expired was 8.68 years while the mean age of patients who survived was 5.54 years. Significantly higher number of patients expired in the age groups of 11–15 years (50.5%) and 16–18 years (57.5%) (*p* < 0.0005). Mortality was significantly higher in females (38.6%) as compared to male children in whom mortality was 26.3% (*p* = 0.004).

**Table 1:** Distribution of patient characteristics between deceased and survivors

		Mortality		Total	OR (95% CI)	*p* value
		Expired	Survived			
Age distribution	<1 year	5 (17.86%)	23 (82.14%)	28 (100.00%)	1.000	
	1–5 years	53 (22.27%)	185 (77.73%)	238 (100.00%)	1.232 (0.457–3.326)	0.680
	6–10 years	22 (28.21%)	56 (71.79%)	78 (100.00%)	1.702 (0.586–4.936)	0.328
	11–15 years	46 (50.55%)	45 (49.45%)	91 (100.00%)	4.367 (1.558–12.235)	0.005
	16–20 years	23 (57.50%)	17 (42.50%)	40 (100.00%)	5.738 (1.847–17.827)	0.003
Total		149 (31.37%)	326 (68.63%)	475 (100.00%)		
Gender	Male	74 (26.33%)	207 (73.67%)	281 (100%)	1.000	
	Female	75 (38.66%)	119 (61.34%)	194 (100%)	1.760 (1.190-2.606)	0.005
Total		149 (31.37%)	326 (68.63%)	475 (100%)		
TBSA %	0–25%	6 (2.75%)	212 (97.25%)	218 (100.00%)	1.000	
	26–50%	48 (32.88%)	98 (67.12%)	146 (100.00%)	16.097 (6.846–37.847)	<0.001
	51–75%	53 (76.81%)	16 (23.19%)	69 (100.00%)	106.002 (40.602–276.745)	<0.001
	76–100%	42 (100%)	0 (0.00%)	42 (100.00%)	2778.855 (148.754–51,911.6)	<0.001
Total		149 (31.37%)	326 (68.63%)	475 (100.00%)		
Depth	Full	16 (39.02%)	25 (60.98%)	41 (100.00%)	2.207 (1.092–4.377)	0.028
	Mixed	80 (40.20%)	119 (59.80%)	199 (100.00%)	2.298 (1.521–3.496)	0.016
	Partial	53 (22.55%)	182 (77.45%)	235 (100.00%)	1.000	
Total		149 (31.37%)	326 (68.63%)	475 (100.00%)		
Mode of injury	Accidental	138 (29.81%)	325 (70.19%)	463 (100.00%)	1.000	
	Homicidal	1 (50.00%)	1 (50.00%)	2 (100.00%)	2.350 (0.190–29.127)	0.467
	Not specified	3 (100.00%)	0 (0.00%)	3 (100.00%)	16.451 (1.580–2217.575)	0.016
	Suicidal	7 (100.00%)	0 (0.00%)	7 (100.00%)	35.253 (4.248–4586.876)	<0.001
Total		149 (31.37%)	326 (68.63%)	475 (100.00%)		
Type of burn	Chemical	0 (0.00%)	3 (100.00%)	3 (100.00%)	1.000	
	Electric	11 (32.35%)	23 (67.65%)	34 (100.00%)	3.426 (0.294–475.488)	0.368
	Scald	42 (18.10%)	190 (81.90%)	232 (100.00%)	1.562 (0.147–211.471)	0.757
	Thermal	96 (46.60%)	110 (53.40%)	206 (100.00%)	6.113 (0.582–825.884)	0.147
Total		149 (31.37%)	326 (68.63%)	475 (100.00%)		
Type of referral	Direct	70 (28.00%)	180 (72.00%)	250 (100.00%)	1.000	
	Referred	79 (35.11%)	146 (64.89%)	225 (100.00%)	1.389 (0.943–2.050)	0.096
Total		149 (31.37%)	326 (68.63%)	475 (100.00%)		
Family type	Joint	41 (43.62%)	53 (56.38%)	94 (100.00%)	1.000	
	Nuclear	108 (28.35%)	273 (71.65%)	381 (100.00%)	0.511 (0.322–0.814)	0.005
Total		149 (31.37%)	326 (68.63%)	475 (100.00%)		
House location	Rural area	59 (41.26%)	84 (58.74%)	143 (100.00%)	1.000	
	Urban area	90 (27.11%)	242 (72.89%)	332 (100.00%)	0.530 (0.352–0.800)	0.003
Total		149 (31.37%)	326 (68.63%)	475 (100.00%)		
Wound culture	Growth	64 (24.24%)	200 (75.76%)	264 (100.00%)	4.182 (1.528–11.443)	
	No growth	4 (6.45%)	58 (93.55%)	62 (100%)	1.000	0.005
Total		68 (20.86%)	258 (79.14%)	326 (100.00%)		
Inhalation injury	YES	55 (74.32%)	19 (25.68%)	74 (100%)	9.261 (5.349–16.646)	<0.001
	NO	94 (23.44%)	307 (76.56%)	401 (100%)	1.000	
		149 (31.37)	326 (68.63%)	475 (100%)		
Total						
Hospital days	<1	14 (93.33%)	1 (6.67%)	15 (100.00%)	1.000	
	1–10	103 (33.99%)	200 (66.01%)	303 (100.00%)	0.053 (0.009–0.308)	0.001
	11–20	17 (15.18%)	95 (84.82%)	112 (100.00%)	0.019 (0.003–.116)	<0.001
	>30	15 (33.33%)	30 (66.67%)	45 (100.00%)	0.053 (0.008–0.332)	0.002
Total		149 (31.37%)	326 (68.63%)	475 (100.00%)		
Month of admission	1) January–March	40 (29.20%)	97 (70.80%)	137 (100.00%)	1.000	
	2) April–June	49 (42.98%)	65 (57.02%)	114 (100.00%)	1.819 (1.084–3.072)	0.024
	3) July–September	50 (40.32%)	74 (59.68%)	124 (100.00%)	1.632 (0.980–2.732)	0.060
	4) October–December	10 (10.00%)	90 (90.00%)	100 (100.00%)	0.279 (0.128–0.565)	<0.001
Total		149 (31.37%)	326 (68.63%)	475 (100.00%)		

*OR* odds ratio, *CI* confidence interval, *TBSA* total body surface area

Higher mortality was seen in patients belonging to joint family where 41 out of 94 patients (43.6%) did not survive as compared to patients from nuclear family where 108 out of 381 patients (28.3%) expired. This association was found to be statistically significant (*p* = 0.04). There was also significant difference (*p* = 0.002) between the mortality rates of children from rural areas (41.2%) and urban areas (27.1%).

**Fig. 1:**
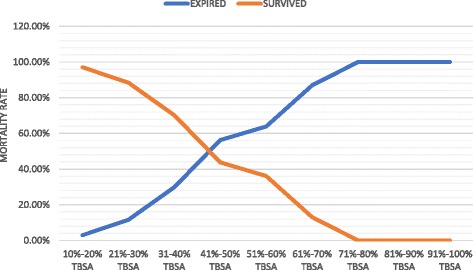
Effect of TBSA on mortality of pediatric burns. Mortality increases significantly with the increase in TBSA. Mortality rate was 100% in patients with TBSA involvement of >70%. *TBSA* total body surface area

The mean TBSA (62%) of the patients who expired was significantly higher (*p* < 0.001) than TBSA (25%) of survivors. The mortality rate increased significantly with increased TBSA. Mortality was 100% in patients with TBSA involvement of 70% or more (*p* < 0.001) [Fig. 1].

Higher mortality rate was observed in full thickness (*n* = 41) and mixed thickness burns (*n* = 199) where it was 39% (*n* = 16) and 40.2% (*n* = 80), respectively. Mortality was lower (22.5%) in partial thickness burns (*n* = 235) where only 53 patients expired (*p* < 0.001).

Of the 34 patients that suffered electric burns, 11 died (32.4%) and among 232 scald burn patients, 43 (18.5%) patients died. Mortality was highest in thermal burns where 96 out of 206 (46.6%) patients died (*p* < 0.001).

Inhalational injury was seen in 15.6% (*n* = 74) of pediatric burn admissions. Mortality was significantly higher (74.3%) in this group as compared to 23.4% in patients that did not have inhalational injury (*p* < 0.001).

The infection rate among expired patients and survivors were 94.1% (64 out of 68) and 77.5% (200 out of 258), respectively. Mortality was highest in patients with positive cultures of *Acinetobacter* + *Klebsiella* (58.3%), followed by *Pseudomonas* + *Klebsiella* (53.3%). Mortality was 33.3, 31.5, and 26.3% in patients with isolates of MRSA, *Acinetobacter*, and of *Pseudomonas*, respectively (*p* < 0.001) [Table 2].

**Table 2:** Mortality observed in various isolates of microorganisms from the wound

	Mortality			
		Expired	Survived	Total number of isolates
Microbiological Profile	*Acinetobacter* + *Klebsiella*	7 (58.33%)	5 (41.67%)	12 (100.00%)
	*Acinetobacter*	23 (31.51%)	50 (68.49%)	73 (100.00%)
	*Clostridia*	0 (0.00%)	2 (100.00%)	2 (100.00%)
	*Escherichia coli*	0 (0.00%)	18 (100.00%)	18 (100.00%)
	*Klebsiella*	2 (7.14%)	26 (92.86%)	28 (100.00%)
	Mixed	4 (11.76%)	30 (88.24%)	34 (100.00%)
	MRSA	1 (33.33%)	2 (66.67%)	3 (100.00%)
	No growth	4 (6.45%)	58 (93.55%)	62 (100.00%)
	*Pseudomonas* + *Klebsiella*	16 (53.33%)	14 (46.67%)	30 (100.00%)
	*Pseudomonas*	10 (26.32%)	28 (73.68%)	38 (100.00%)
	*Staphylococcus aureus*	1 (3.85%)	25 (96.15%)	26 (100.00%)
Total		68 (20.86%)	258 (79.14%)	326 (100.00%)

*MRSA* methicillin resistant *Staphylococcus aureus*

**Fig. 2:**
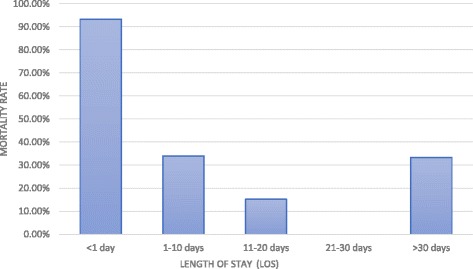
Association between length of stay (LOS) in the hospital and pediatric burn mortality. Mortality was highest in patients with LOS of <1 day (93.33%). Mortality rate was 0% in patients who stayed in the hospital for 21–30 days

The mean length of stay (LOS) in the hospital was 9.94 + 7.57 days. Mean LOS in the hospital was 7.42 days among 149 patients that expired while it was 11.1 days in case of survivors. There was no mortality in patients who stayed in the hospital for 21–30 days (*p* < 0.001) [Fig. 2].

**Fig. 3:**
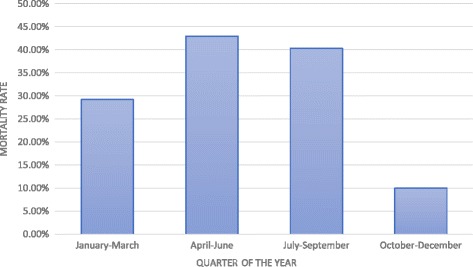
Mortality rates observed in different quarter of the year. Pediatric burn mortality was lowest (10%) in patients admitted during the last quarter of the year and highest (42.9%) in second quarter of the year (*p* = <0.001)

Higher mortality was observed in patients admitted between the months of April–June when 49 out of 114 admitted (42.9%) patients expired. Mortality was lowest in patients admitted between the months of October to December (10%) (*p* < 0.001) [Fig. 3].

### Firth logistic regression

Odds ratio (OR) based on firth logistic regression and 95% confidence interval (CI) was estimated for a number of risk factors based on above analysis. Variables with *p* value of <0.05 on univariate regression were included in the multivariate regression model. Multicollinearity was checked by calculating variance inflation factor (VIF). As none of the variables had VIF > 5, the problem of multicollinearity did not exist. On univariate firth analysis, age > 11 years [OR (95% CI) = 4.367 (1.558–12.235)], female gender [OR (95% CI) = 1.760 (1.190–2.606)], suicidal burns [OR(95%CI) =35.253 (4.248–4586.876)], TBSA of >25% [OR(95% CI) = 16.097 (6.846–37.847)], inhalation injury [OR (95% CI) = 9.261 (5.349–16.646)], deeper burns [OR (95% CI) =2.207 (1.092–4.377)], and positive wound culture [OR (95% CI)] = 4.182 (1.528–11.443)] were found to be the significant factors for increased mortality [Table 1]. Significantly decreased risk of mortality was seen in patients belonging to urban areas [OR (95% CI) = 0.530 (0.352–0.800)], nuclear families [OR (95% CI) = 0.511 (0.322–0.814)], and in patients who were admitted during October to December [OR (95% CI) = 0.279 (0.128–0.565)].

After adjusting for confounding factors and performing multivariate firth logistic regression, higher TBSA was identified as an independent risk factor for mortality in pediatric burns. Risk of mortality was significantly higher in patients with TBSA involvement of 25.1–50% [OR (95% CI) − 21.706 (6.489–72.608)], 50.1–75% [OR (95% CI) − 136.195 (31.157–595.345)], and 75.1–100% [OR (95% CI) − 1019.436 (26.795–38,784.79)] [Table 3].

**Table 3:** Results from multivariate firth analysis predicting in-hospital mortality in pediatric burns

	Adjusted odds ratio (95% CI)	*p* value	Overall likelihood ratio	R squared
Age				
<1 year	1.000			
1–5 years	1.299(0.228–7.406)	0.768		
6–10 years	1.215 (0.187–7.915)	0.838		
11–15 years	0.545 (0.081–3.65)	0.532		
16–20 years	0.346 (0.039–3.036)	0.338	116.21	0.76
TBSA%				
1) 0–25%	1.000			
2) 25.1–50%	21.706 (6.489–72.608)	<0.0001		
3) 50.1–75%	136.195 (31.157–595.345)	<0.0001		
4) 75.1–100%	1019.436 (26.795–38,784.79)	0.0002		
Hospital days				
<1	1.000			
1–10	11.844 (0.081–1734.005)	0.331		
11–20	2.292 (0.016–325.122)	0.743		
>20	2.299(0.017–307.916)	0.739		
Gender				
Male	1.000			
Female	0.962 (0.455–2.035)	0.920		
Inhalation injury	1.937 (0.663–5.663)	0.227		
Depth				
Partial	1.000			
Mixed	1.283 (0.582–2.828)	0.537		
Full	0.538 (0.126–2.303)	0.404		
Burn cause				
Accidental	1.000			
Homicidal	19.724 (0.131–2973.145)	0.2439		
Suicidal	16.271 (0.112–2361.275)	0.2720		
Family type				
Joint	1.000			
Nuclear	0.895 (0.368–2.18)	0.808		
House location				
Rural area	1.000			
Urban area	0.488 (0.232–1.025)	0.058		
Month of admission				
January–March	1.000			
April–June	0.61 (0.22–1.69)	0.342		
July–September	0.591 (0.217–1.612)	0.304		
October–December	0.327 (0.105–1.022)	0.055		
Growth on wound culture	2.907 (0.611–13.837)	0.180		

## Discussion

Our previous study described an epidemiology of pediatric burn in north India and presented various preventive strategies to decrease the incidence of burn-related injuries in children [19]. Whereas, the present study reports on the in-hospital mortality of burn injuries in children and aimed to analyze the various risk factors associated with mortality in pediatric burns.

Analysis of mortality data and application of multivariate logistic regression model demonstrated TBSA as the strongest predictor of mortality in our pediatric burn patients. Risk of mortality increases 21 times for TBSA of 25–50% and 136 times for TBSA of 50–75%. This is consistent with the findings of multiple studies worldwide, in both pediatric and adult population [9, 20–23]. The role of higher TBSA exponentially affecting the mortality in children is firmly established by this study. No other factor was independently responsible for increased mortality in pediatric burns after adjusting for confounding factors.

Many studies have demonstrated the increased risk of mortality in children up to the age of 5 years as compared to older children [3, 20–22, 24–26]. A study by Wolf et al. demonstrated age as a significant predictor of mortality even in massively (>70% TBSA) burn children [27]. In contrast, we found that risk of mortality is higher in older children (age > 11 years). An analysis of this group of patients showed that these children had higher incidence of thermal burns (*n* = 102; 77.8%), inhalation injury (*n* = 46; 35.1%), and higher TBSA (mean TBSA = 56.5%) that contributed to higher mortality in this subset of patients. We have previously reported that TBSA involved increases with increase in age [19].

Female gender, presence of inhalation injury and deeper burns were found to be significantly affecting pediatric burn mortality on univariate regression analysis. Gender bias could be explained by significantly higher TBSA (mean 43 ± 28%) involvement in female patients against the overall mean of 37%. A study from Tanzania on predictors of pediatric burn mortality had reported female gender association with mortality that showed trend towards being significant [28]. Similar association was not found in other studies [26, 29]. Females take up the task of cooking in the kitchen at an early age making them more prone to fire-related injuries. Likewise, the widespread usage of kerosene stoves rather than liquified petroleum gas (LPG) compromises the safety of these children in kitchen.

We observed a high infection rate of 80.9% at our burn center (77.5% in survivors and 94.1% in expired patients). Other burn centers from India also reports similarly high infection rates [30–32]. This points out to the difference in standard of care of burn injuries in low and medium income﻿ countries to high income countries. The reasons for decreasing infection rate in developed countries are their focus on developing effective prevention strategies and early wound coverage. These measures reduced their sepsis-related mortality from 14% to 3% in 5 years period [33]. In contrast, the standard of care in developing countries like India is conservative management, instead of early excision and grafting. The preventive strategies to reduce high infection rate in pediatric burns include: early referral of the patient to burn center, preventing microbial colonization of wound by maintaining strict sterility, early coverage of wound, and optimal nutritional support to the patient.

Some studies had identified association of invasive procedures such as catheters with increased risk of infections and mortality. Blood stream infection in burn patients had also been found to be associated with mortality [34, 35]. Pseudomonas and Acinetobacter were the most common isolates from different sources in a study from Brazil where authors found significant association between infections and mortality [20]. We found positive wound cultures of microorganisms to be a significant risk factor (OR = 4.182) for mortality on univariate logistic regression analysis. All of these microorganisms cause gram-negative septicemia leading to pulmonary and renal complications and subsequently multiple organ dysfunction. Emergence of multi resistant strains continues to be a challenge in the management of pediatric burn patients.

Inhalation injury has been traditionally associated with higher mortality in burn injuries. There are studies implicating inhalation injury as an independent risk factor for mortality in children and when associated with other risk factors, it significantly increases the mortality from burn injuries [11, 23, 29]. Our cohort also had similar association between inhalational injury and patient mortality. Most of these patients developed pulmonary complications and required mechanical ventilation.

Few studies had reported higher incidence of mortality in patients sustaining thermal burns as compared to scald injuries. Higher mortality among thermal burn patients is the result of higher TBSA involvement, greater depth of burns, and associated inhalational injury [36–38]. However, we did not find any evidence incriminating thermal burns as a significant risk factor for mortality in pediatric burns.

Interesting part of the outcomes from this study was the identification of factors decreasing the risk of mortality in burn children, as observed by negative standardized beta coefficient. Admissions in the last quarter of the year (October to December) were found to be associated with significantly decreased mortality. On careful analysis, we found that majority of the patients admitted during this period had less TBSA involvement (mean TBSA = 30% + 18%) and decreased instances of thermal and inhalation injuries. Lesser environmental temperatures can be another reason for the above observation as these are winter months in New Delhi with temperature dipping up to subzero levels.

An Australian study attempted to determine the differences between the burn characteristics of children from rural and urban areas. Although there were significant differences between cohort characteristics of rural and urban children, there was no significant difference in the mortality rate of these children [39]. However, on univariate regression analysis, our study demonstrated significantly decreased mortality in children who belonged to nuclear families and in those who were from urban areas. This implies that people from urban areas are more aware of the needs of patients, and they are swift in seeking health care unlike patients from rural areas where delay in arrival at a tertiary care center is common.

Average LOS for our patients was 9.95 days. Mortality was found to decrease with increasing LOS in our patient cohort. No other study demonstrated the relationship between LOS and mortality in children. Majority of patients who died in the first few days either had extensive burns with inhalational injuries or reached hospital late without any first aid. Patients who expired after 30 days were severely catabolic and developed sepsis with multi organ dysfunction.

Likelihood of mortality is one of the means of looking at the results between different burn centers [18]. Regardless of the advances in critical care medicine and burn ward management, the mortality is high in developing countries. We observed a mortality rate of 31.3% that is very high in contrast to other studies from India that reported a mortality rate of 7–12% [1, 2, 4]. A study from Nigeria had reported mortality rate for thermal burns as 35.6% and 18.9% for scalds [38]. Higher mortality rate in our burn unit might be attributed to low frequency of early excision and grafting among our patients, large number of admissions with higher TBSA burn, delayed arrival from referral centers, inadequate treatment during referral, higher number of thermal burns with or without inhalational injuries, and prevalence of multi drug resistant Acinetobacter, Pseudomonas, and Klebsiella species. It may be possible that being a tertiary care burn center, only those patients with little chances of survival are referred to our center after triage at lower centers that might have contributed to higher mortality observed in this study.

The most effective strategy to decrease burn-related deaths in children is to apply effective preventive measures that reduce the incidence of burn-related injuries. Primary excision and grafting is the standard of care for burns today. As the donor area is limited in children, other means are required to cover the burn wound as early as possible. These include various biological and non-biological skin substitutes. In a country with limited resources, the availability of these materials is scant especially in government hospitals. Requirement for the widespread availability of biotechnology methods and bioengineering facilities (e.g., laboratories for keratinocyte cultures and development of skin substitutes) is the need of an hour to decrease the burn-related deaths in children from developing countries.

## Conclusion

This study demonstrated that TBSA is the only risk factor that independently affects the mortality in pediatric burns. The risk of mortality significantly increases with increased TBSA. Other risk factors significantly affecting mortality are age > 11 years, female gender, deeper burns, inhalation injury, and positive wound cultures. Mortality was significantly lower in children from urban areas, nuclear families, and in those who stayed in the hospital for longer period. Patients admitted during the last quarter of the year were at decreased risk for mortality from burn injuries. Finally, we need to acknowledge the presence of high mortality rate among pediatric burn patients in our country and efforts should be directed to reduce it by targeting children with above mentioned risk factors.

## Abbreviations

BICU: Burn intensive care unit
CI: Confidence interval
LOS: Length of stay
MRSA: Methicillin resistant *Staphylococcus aureus*
OR: Odds ratio
SPSS: Statistical Package for Social Sciences
TBSA: Total body surface area
VIF: Variance inflation factor

## References

[1] Kumar P, Chirayil PT, Chittoria R (2000). Ten years epidemiological study of paediatric burns in Manipal, India. Burns.

[2] Mukerji G, Chamania S, Patidar GP, Gupta S (2001). Epidemiology of pediatric burns in Indore, India. Burns.

[3] Gupta M, Gupta OK, Yaduvanshi RK, Upadhyaya J (1993). Burn epidemiology: the Pink City scene. Burns.

[4] Burd A, Yuen C (2005). A global study of hospitalized paediatric burn patients. Burns.

[5] Ramakrishnan KM, Jayaraman V, Mathivanan T, Babu M, Ramachandran B, Sankar J (2012). Profile of burn sepsis challenges and outcome in an exclusive children’s hospital in Chennai, India. Ann Burns Fire Disasters.

[6] Baux S. Contribution a l’etude du traitement local des brulures thermiques etendues. Paris: These; 1961.

[7] Clark CJ, Reid WH, Gilmour WH, Campbell D (1986). Mortality probability in victims of fire trauma: revised equation to include inhalation injury. Br Med J.

[8] Gomez M, Wong DT, Stewart TE, Redelmeier DA, Fish JF (2008). The FLAMES score accurately predicts mortality risk in burn patients. J Trauma.

[9] Moore EC, Pilcher DV, Bailey MJ, Cleland H, Stevens H (2013). A simple tool for mortality prediction in burns patients: APACHE III score and FTSA. J Trauma.

[10] Tsurumi A, Que YA, Yan S, Tompkins RG, Rahme LG, Ryan CM (2015). Do standard burn mortality formulae work on a population of severely burned children and adults?. Burns.

[11] Saeman MR, Hodgman EI, Burris A, Wolf SE, Arnoldo BD, Kowalske KJ (2016). Epidemiology and outcomes of pediatric burns over 35 years at parkland hospital. Burns.

[12] Xu JH, Qiu J, Zhou JH, Zhang L, Yuan DF, Dai W (2014). Pediatric burns in military hospitals of China from 2001 to 2007: a retrospective study. Burns.

[13] Li H, Wang S, Tan J, Zhou J, Wu J, Luo G. Epidemiology of pediatric burns in southwest China from 2011 to 2015. Burns. 2017; 10.1016/j.burns.2017.03.004. 10.1016/j.burns.2017.03.004 28372828

[14] John TJ (1999). Indian association of pediatrics (IAP) policy on age of children for pediatric care. Indian Pediatr.

[15] Sahu SA, Agrawal K, Patel PK (2016). Scald burn, a preventable injury: analysis of 4306 patients from a major tertiary care center. Burns.

[16] Chauhan N, Kumar S, Sharma U (2012). Profile of acute thermal burn admissions to the intensive care unit of a tertiary burn care center in India. Indian J Burns..

[17] Sharma RK, Parashar A (2010). Special considerations in paediatric burn patients. Indian J Plast Surg.

[18] Greenhalgh DG, Saffle JR, Holmes JH, Gamelli RL, Palmieri TL, Horton JW (2007). American burn association consensus conference to define sepsis and infection in burns. J Burn Care Res.

[19] Dhopte A, Tiwari VK, Patel P, Bamal R (2017). Epidemiology of pediatric burns and future prevention strategies—a study of 475 patients from a high-volume burn center in North India. Burns Trauma.

[20] Taylor SL, Lawless M, Curri T, Sen S, Greenhalgh DG, Palmieri TL (2014). Predicting mortality from burns: the need for age-group specific models. Burns.

[21] Rosanovaa MT, Stamboulianb D, Ledeca R (2014). Risk factors for mortality in burn children. Braz J Infect Dis.

[22] Agbenorku P, Agbenorku M, Fiifi-Yankson PK (2013). Pediatric burns mortality risk factors in a developing country’s tertiary burns intensive care unit. Int J Burns Trauma.

[23] Benito-Ruiz J, Navarro-Monzonis A, Baena-Montilla P, Mirabet-Ippolito V (1991). An analysis of burn mortality: a report from a Spanish regional burn centre. Burns.

[24] Morrow SE, Smith DL, Cairns BA, Howell PD, Nakayama DK, Peterson HD (1996). Etiology and outcome of pediatric burns. J Pediatr Surg.

[25] Sheridan RL (1998). The seriously burned child: resuscitation through reintegration-1. Curr Probl Pediatr.

[26] Dermijian G (1997). Adjusting a prognosis score for burned children with logistic regression. J Burn Care Rehabilit.

[27] Wolf SE, Rose JK, Desai MH, Mileski JP, Barrow RE, Herndon DN (1997). Mortality determinants in massive pediatric burns. Annals Surg.

[28] Chelidze KI, Lim CC, Peck RN, Giiti G, Leahy N, Rabbitts A (2016). Predictors of mortality among pediatric burn patients in East Africa. J Burn Care Res..

[29] Barrow RE, Spies M, Barrow LN, Herndon DN (2004). Influence of demographics and inhalation injury on burn mortality in children. Burns.

[30] Bhatt P, Rathi KR, Hazra S, Sharma A, Shete V (2015). Prevalence of multidrug resistant Pseudomonas Aeruginosa infection in burn patients at a tertiary care centre. Indian J Burns..

[31] Khan TS, Bijli AH, Wani AH (2016). Microbiological and quantitative analysis of burn wounds in the burn unit at a tertiary care hospital in Kashmir. Indian J Burns..

[32] Mohapatra S, Deb M, Agrawal K, Chopra S, Gaind R (2014). Bacteriological profile of patients and environmental samples in burn intensive care unit: a pilot study from a tertiary care hospital. Indian J Burns.

[33] Murray CK, Loo FL, Hospenthal DR, Cancio LC, Jones JA, Kim SH (2008). Incidence of systemic fungal infection and related mortality following severe burns. Burns.

[34] Sheridan RL (2005). Sepsis in pediatric burn patients. Pediatr CritCare Med.

[35] Derganc M (1993). Present trends in fluid therapy, metabolic care, and prevention of infection in burned children. Crit Care Med.

[36] Torabian S, Sadegh SM (2009). Epidemiology of paediatric burn injuries in Hamadan, Iran. Burns.

[37] Goldman S, Aharonson-Daniel L, Peleg K, Israel Trauma Group (ITG) (2006). Childhood burns in Israel: a seven year epidemiological review. Burns.

[38] Fadeyibi IO, Mustapha IA, Ibrahim NA, Faduyile FI, Faboya MO, Jewo PI (2011). Characteristics of paediatric burns seen at a tertiary centre in a low income country: a five year (2004-2008) study. Burns.

[39] Hyland EJ, Zeni G, Harvey JG, Holland AJ (2015). Rural and metropolitan pediatric burns in new South Wales and the Australian Capital Territory: does distance make a difference?. J Burn Care & Res.

